# The Role of the Carotid Doppler Examination in the Evaluation of Atherosclerotic Changes in *β-*Thalassemia Patients

**DOI:** 10.4084/MJHID.2015.023

**Published:** 2015-03-01

**Authors:** Hoda A. Abdelsamei, Ashraf M. El-Sherif, Ahlam M. Ismail, Gehan L. Abdel Hakeem

**Affiliations:** 1 Diagnostic Radiology Department, South Valley University; 2 Diagnostic Radiology Department and, Minia University; 3 Pediatrics Department South Valley University; 4 Pediatrics Department Minia University Egypt

## Abstract

**Background:**

Iron overload in patients with beta-thalassemia major (BTM) lead to alterations in the arterial structures and the thickness of the carotid arteries. Doppler ultrasound scanning of extra-cranial internal carotid arteries is non-invasive and relatively quick to perform and may identify children at increased risk of stroke that would otherwise be missed. Increased carotid artery intima media thickness (CIMT) is a structural marker for early atherosclerosis and correlates with the vascular risk factors and to the severity and extent of coronary artery disease.

**Objective:**

To evaluate the role of carotid Doppler examination and CIMT measurement as a predictor of atherosclerotic changes in BTM children with iron overload.

**Patients and Methods:**

Sixty two children with BTM and, thirty age and sex matched normal controls were included. Complete blood count, ferritin, serum cholesterol were done, as well as carotid Doppler ultrasonography to measure the CIMT in both patients and controls.

**Results:**

CIMT of thalassemic patients was significantly increased compared to controls (*p*=0.001). There was a significant positive correlation between CIMT and patient’s age, the duration from first blood transfusion, serum cholesterol and, iron overload parameters as serum ferritin, frequency of blood transfusion, iron chelation. The length of the transfusion period was the highest risk factor, and an inadequate iron chelation was a further risk factor. Significant negative correlation was found between CIMT and hematocrit value while no significant correlation was found between CIMT and weight, height, BMI centiles and Hb level.

**Conclusion:**

Carotid Doppler is very useful in measurement of CIMT that increased in thalassemic patients that shows a strong relationship with features of iron overload. Routine Doppler measurement of CIMT in these patients is recommended to predict early atherosclerotic changes as well as in follow-up.

## Introduction

Thalassemia syndromes are groups of heterogeneous hereditary diseases characterized by a decrease or a total absence of synthesis of α- and/or β-globin chains composing the hemoglobin protein in red blood cells (RBCs). They are classified according to the type of deficient globin chain as α- and β-thalassemia.^2^ In β-thalassemia, the severity of the pathophysiology depends on the level of β-globin chain deficiency, which leads to an excess of α-globin chains.^3^ Consequently, thalassemic RBCs are hypochromic and microcytic and have a shorter half-life, leading to anemia.^4^ Three clinical phenotypes of decreasing severity have been established: a transfusion-dependent state, thalassemia major, a moderate phenotype, thalassemia intermedia, and a benign heterozygous condition, thalassemia minor. The patients display heterogeneous clinical and laboratory features, besides several systemic manifestations.^5^ According to the so called “iron hypothesis,” iron is believed to be detrimental for the cardiovascular system, thus promoting atherosclerosis development and progression.^6^ Iron over-load in patients with beta-thalassemia major lead to alterations in the arterial structures and the thickness of the carotid arteries. In addition, carotid thickness positively correlated with age, Hb, ferritin and cholesterol levels in these patients. As a result, coronary artery diseases (CAD) is a quite common cardiovascular complication in thalassemics. Patients on a regular transfusion regimen progressively develop clinical manifestations of iron overload associated with heart dysfunction and left ventricular failure if inadequately chelated.^7^ Doppler ultrasound scanning of extracranial internal carotid arteries is non-invasive and relatively quick to perform and may identify children at increased risk of stroke who would otherwise be missed.^8^ High resolution ultrasound is a reliable, method for detecting early structural and functional atherosclerotic changes in the arterial wall.^9^ Increased carotid artery intima media thickness (CIMT) is a structural marker for early atherosclerosis, and it correlates with the vascular risk factors and to the severity and extent of coronary artery disease.(10,11)

The study objectives: This study aimed to evaluate the role of carotid Doppler examination and CIMT measurement as a predictor to atherosclerotic changes in B thalassemia major (BTM) children with iron overload.

## Patients and Methods

This is a cross sectional case control study. Sixty two children with BTM were selected from the pediatric hematology outpatient clinics in Qena and Menia University hospitals as well as 30 healthy normal age and sex matched controls in the period from May 2013 to September 2014. Diagnostic criteria, assessment and management of thalassemic children were based on Guidelines for the Clinical Management of Thalassemia.^12^ The study was approved by the local research ethics committee of the two hospitals and written informed consent was obtained from the parents of all children to share in the study. Included patients with B thalassemia proved by clinical and laboratory investigations, frequent blood transfusion, chelation therapy. Patients with familiar hypercholesterolemia (confirmed by history), cardiovascular symptoms suggesting the presence of heart failure or atherosclerotic changes and patients with chronic systemic illness were excluded. All patients were subjected to the following work-up assessment:

- The history including the duration of the illness since the first blood transfusion, the frequency of blood transfusion (frequent ≥ 2 times/month) and the intake of iron chelating agents. Chelation therapy was initiated when serum ferritin levels reached approximately 1000 ng/mL (subcutaneous deferroxamine, oral deferopron or combination of both). Patients were classified as adequately chelated, poorly chelated or non-chelated according to serum ferritin level, frequency of blood transfusion and regularity of chelation.
- Clinical examination including general, chest, heart and abdominal examination. Patient’s anthropometric measurements were plotted on growth charts (Official 2000 centers for centers for disease control (CDC) growth charts, created by the National Center for Health Statistics (NCHS).^13^
- Laboratory and radiological investigations including complete blood count, hemoglobin electrophoresis, serum levels of ferritin and iron and iron binding capacity all were done at the time of the study. Abdominal ultrasound was performed for detection of the hepatomegaly and/or splenomegaly.

Carotid duplex study: All patients and controls were subjected to B-mode and color-coded duplex sonography of their extra-cranial carotid and vertebral arteries. All studies were performed using a LOGIC P6 ultrasound system (GE medical systems, Milwaukee, WI) with a 12.0-MHz linear array transducer. All ultrasound examinations were performed by a single experienced vascular radiologist who was unaware of the clinical and laboratory details of the examined children. Examination started by locating the common carotid artery (CCA) in the lower neck in the transverse plane. The CCA is followed proximally until the transducer is blocked by the clavicle, and caudal angulations is tried to evaluate the common carotid origin if possible. The CCA continues upwards till it widens to form the carotid bulb; then it bifurcates into internal and external branches. The transducer is then rotated 90 degrees to be parallel to the CCA to have longitudinal scanning of the CCA, the bifurcation, the internal carotid artery (ICA) and external carotid artery (ECA). The ICA was then followed distally as far as possible and optimally until it is lost behind the mandible. The vessels were evaluated meticulously for the presence of subintimal lucency, and atherosclerotic plaques that bulge into the lumen, followed by measuring the intimal plus medial thickness (IMT). IMT was measured in 1-cm segment proximal to the dilation of the carotid bulb, referred to as CCA, and always in plaque-free segments. For each subject, three measurements on both sides were obtained on the anterior, lateral, and posterior projection of the far wall. Values for the different projections and right and left arteries were then averaged. Two end-diastolic frames were selected and analyzed for mean CIMT, and the average reading from these two frames was calculated for both right and left carotid arteries. The average of the two sides was considered the patient’s overall mean CIMT. Statistical analysis: The data were statistically analyzed using the SPSS software package, version 16 (SPSS Inc., Chicago, IL, USA) on a personal computer. Numerical data were expressed as range, mean± SD, median, and percentiles. Non numerical data were expressed as frequencies. Comparative studies were done using Student t test and chi square test. (p value < 0.05 was considered significant). Pearson correlation test was used to detect correlation between different parameters. In addition, multiple regression analysis was done to identify the most significant risk factors.

## Results

**Figure 1:**
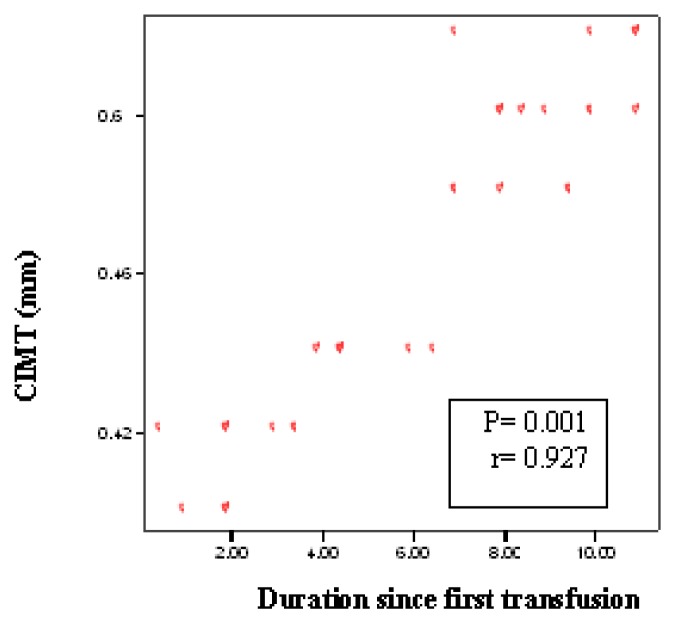
Correlation between CIMT and duration since first transfusion

**Figure 2:**
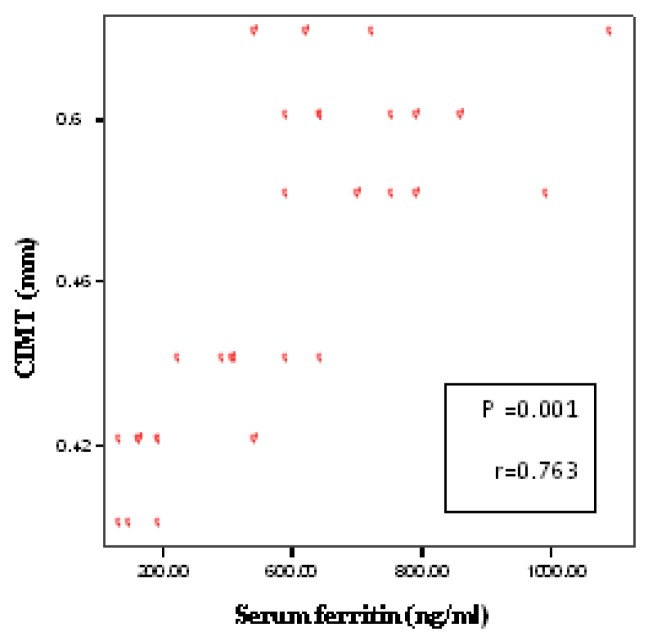
Correlation between CIMT and serum ferritin.

**Figure 3:**
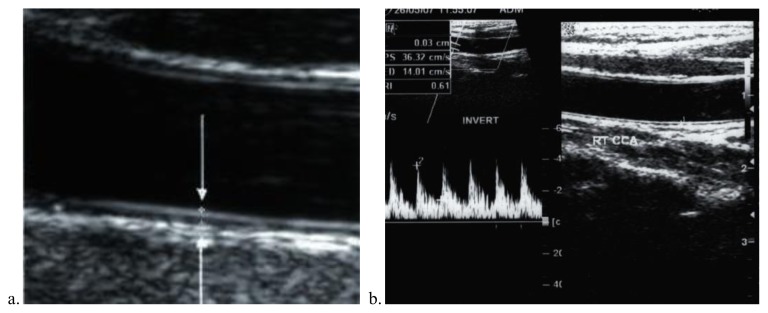
**a)** Long-axis magnified view of the normal carotid wall anatomy on U/S. The intima and adventia produces echogenic parallel lines (arrows) with an intervening echo void representing the media. **b)** Long-axis view and Doppler spectrum of the right CCA showing normal intima-media thickness of 0. 3 mm in a 10-year-old healthy child.

**Figure 4:**
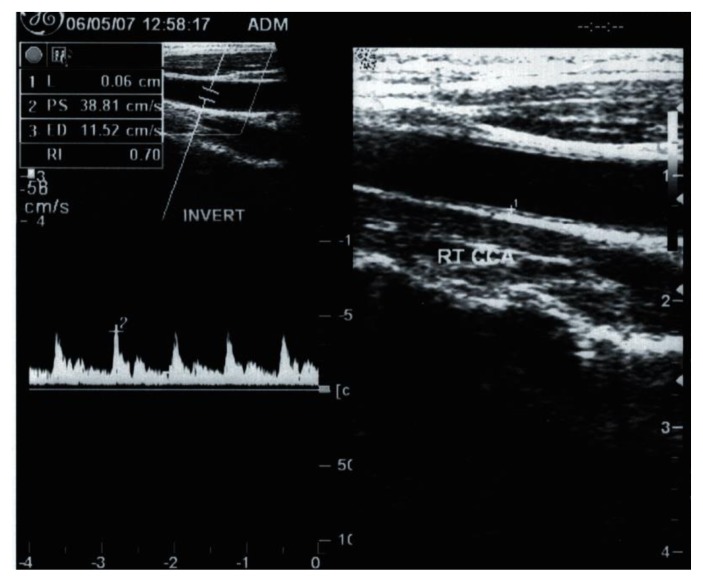
Long-axis view and Doppler spectrum of the right CCA showing increased intima-media thickness of 0.6-mm in an 11-year-old child with thalassemia.

**Figure 5:**
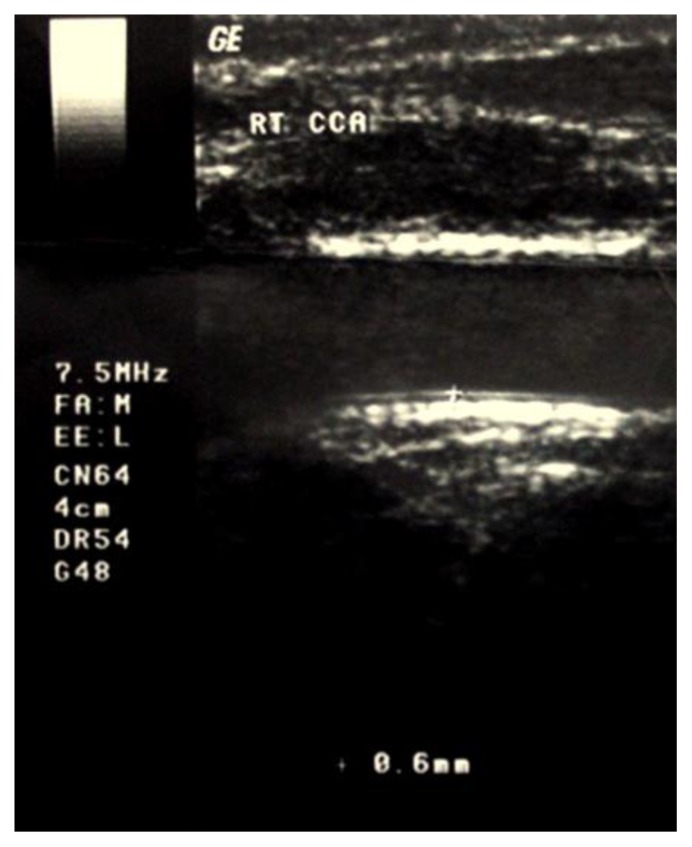
Long-axis view of the right CCA showing increased intima-media thickness of 0.6-mm in a 9-year-old child with thalassemia.

**Figure 6:**
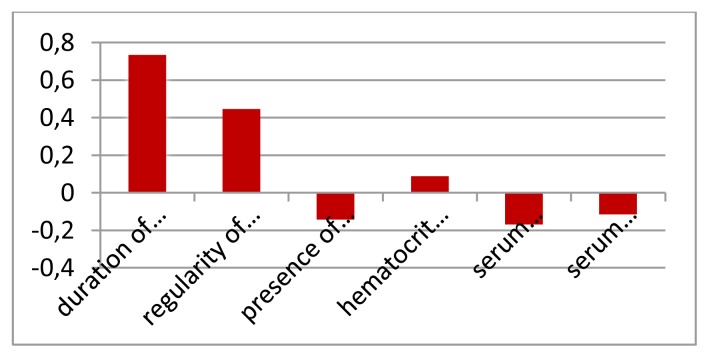
Risk factors of increased CIMT in B thalassemic patients (Multiple regression analysis).

Demographic, clinical and laboratory data for patients and controls are shown in Table 1. No significant difference between patients and controls regarding age or gender, while significant difference was found regarding weight, height and BMI centiles, Hb level, hematocrite value, serum ferritin and serum cholesterol. The duration since first transfusion ranged from 1.5–13 years with a mean of 7.26± 3.7. Thirty two patients (51.6%) had frequent blood transfusion, 24 patients (38.7%) were adequately chelated, and 24 patients (38.7%) undergone splenectomy operation. Table 2 shows comparison between patients and controls regarding CIMT. There was a significant difference between studied patients and controls regarding CIMT measurements (*p* =0.001). As shown in table 3 and figures 1 and 2, significant positive correlation was found between CIMT and patient’s age, duration since the first blood transfusion, serum ferritin and serum cholesterol. Significant negative correlation was found between CIMT and hematocrit value while no significant correlation was found between CIMT regarding weight, height, BMI centiles and Hb level. CIMT was significantly increased in BTM children in relation to children with frequent blood transfusion, patients who were poorly chelated or had splenectomy (Table 4). Figures 3–5 show the ultrasonographic CIMT measurements and the Doppler spectrum of the carotid vessels in healthy controls and in patients with BTM. Table 5 and figure 6 display the risk factors increasing CIMT in thalassemic patients. Duration of illness carries the highest risk factor followed by the inadequate iron chelation therapy.

## Discussion

Beta-thalassemia is a group of hereditary blood disorders first described by Cooley and Lee.^14^ With the increased life span of BTM patients; coronary artery disease may emerge as one of the important cardiovascular complications.^15^ Studies have suggested a link between iron load and risk of atherosclerosis. The present study was undertaken to evaluate the role of carotid Doppler examination and CIMT measurement as a predictive to atherosclerotic changes in BTM children with iron overload. Our data show that the CIMT of thalassemic patients was significantly increased compared to controls. This finding is supported by the results of some previous studies;^16^–^18^ Cheung et al.,^19^ found an increase in the CIMT in patients with BTM compared to controls, Tantawy et al., 2009^17^ and Gullu et al.,^18^ found the same results in their study, and Adly et al.^19^ reported that Carotid IMT measurements were significantly but slightly higher in the BTM group than that in the controls (0.57 ± 0.07 vs. 0.54 ± 0.04, P = 0.04) and that CIMT is increased in patients with BTM. On the other hand, a previous study, carried out by Cusmà et al.,^20^ comparing the CIMT between the thalassemic patients and healthy subjects (0.67 ± 0.20 mm vs. 0.66 ± 0.15 mm), showed no significant difference. The conclusion of this research was that 2-dimensional strain and echo-tracking might be more accurate than standard echocardiography and vascular parameters in the early identification of cardiovascular involvement.

The development of carotid artery wall hypertrophic remodeling, found in thalassemic patients, is characterized by an increase in both total wall thickness and wall-to-lumen ratios; hemolysis likely contributed to the pathophysiology of both endothelial dysfunction as well as vascular, structural and mechanical, changes.^22^ In our study, there was significant statistical correlation between CIMT and patient’s age, ferritin, and total cholesterol levels but there was no significant difference of CIMT in relation to patient’s hemoglobin level. This also comes in harmony with the study of Tantawy et al.^17^ who reported that in thalassemic patients, CIMT was positively correlated with age, ferritin and cholesterol levels, and that atherogenic lipid profiles in young thalassemic patients with increased CIMT highlights their importance as prognostic factors for vascular risk stratification. These findings are further supported by Gursel et al.,^22^ who investigated the relationship between chronic hemolysis and increased body iron burden and the development of premature atherosclerosis by using CIMT, ferritin, serum lipid profile. They concluded that Subclinical atherosclerosis in children with β-thalassemia major begins early in life, and these children are at risk for development of premature atherosclerosis. Iron overload is usually associated with regular blood transfusions that lead to transfusional haemosiderosis in patients with chronic anemia in children with BTM.^23^ These changes occur initially in reticulo-endothelial system and secondary in all parenchymal organs, mainly heart, pancreas, pituitary gland, and gonads, with cytotoxic effects.^24^ So, accumulation of iron has been implicated as a risk of cardiovascular disease, because of the catalytic role of iron, causing oxidative stresses on the vessel wall.^25^–^27^ We also found that CIMT was significantly different in children with BTM in relation to features suggestive of iron overload including duration since first blood transfusion which carries the highest risk factor of increasing CIMT, frequency of blood transfusion, irregular use of iron chelating agents in patients who were poorly chelating or had splenectomy. This comes in harmony with the results of a previous study that was carried out by Cheung et al,^28^ who found that iron overloading in patients with beta-thalassemia major results in alterations of arterial structures with disruption of elastic tissue and calcification. This finding is also supported by Ramakrishna et al.,^29^ as well as other epidemiological studies concluding that iron is an important factor in the process of atherosclerosis^27^ and that CIMT is considered an early marker of atherosclerotic process and is currently used to assess the presence and the progression of atherosclerosis.^30^,^31^ A significant positive correlation was found in this study between duration since first blood transfusion and CIMT, the longer the duration, the more atherogenesis as reflected by increased CIMT. Moreover, also, there was another significant positive correlation between serum ferritin levels and CIMT. Duration of illness carries the highest risk factor of increasing CIMT followed by the iron chelation therapy guided by serum ferritin. The catalytic role of iron in free radical reactions causes oxidation of LDL and may be an important factor in the formation of atherosclerotic lesions. Studies have shown that iron can stimulate lipid peroxidation in vitro and in vivo.^29^ Oxidized LDL is followed by accumulation of lipids in cells and the formation of foam cells, characteristic of early atherosclerosis. Thereby, oxidized LDL has cytotoxic capacity that induces changes in endothelial cells with loss of endothelial integrity, which could facilitate further accumulation of both circulating monocytes and LDL and thus promote the progression of the atherosclerotic lesion.^32^^–35^

## Conclusion

Carotid Doppler is very useful method in measurement of CIMT that increased in thalassemic patients. CIMT shows a strong relationship with features of iron overload and atherosclerotic changes. Duration of illness carries the highest risk factor of increasing CIMT followed by inadequate iron chelation therapy. Prevention of the progression of atherosclerosis in early stages is important by decreasing body iron load in the thalassemic patients. We recommend the routine use of Doppler measurement of CIMT in BTM patients as a non-invasive diagnostic method to predict early atherosclerotic changes as well as in the follow-up to prevent progression of atherosclerosis. Reducing hyper-lipidemia and body iron load in the thalassemic patients by dietary restriction or pharmacological therapy and good compliance of iron chelating agents is also recommended.

## Figures and Tables

**Table 1:** Demographic, clinical and laboratory data for studied patients and control.

Parameter		Patients (No: 62)	Control (N: 30)	P value#

**Age (years)**	**Range**	3–14 years	3–13	0.628
	**mean± SD**	8.05±3.12	6.5±3.5	

**Gender**	**Male**	34 (54.8%)	19 (63.3%)	0.442
	**Female**	28 (45.2%)	11 (36.7%)	

**Weight (kg) for age centile**	**Range**	5^th^–50th	25^th^–90^th^	0.025*
	**mean± SD**	23.4±15.2	48±20.95	
	**Median**	21	25	

**Height (cm) for age centile**	**Range**	5^th^–90^th^	25^th^–90^th^	0.001*
	**mean± SD**	35.8±25.4	47.17±15.52	
	**Median**	111	114	

**BMI (kg/m^2^) centile**	**Range**	3–97	10^th^–97^th^	0.001*
	**mean± SD**	31.1±37.7	68.4±22.15	
	**Median**	14.51	16.3	

**Duration since the 1^st^** **transfusion (years)**	**Range**	1.5–13	--------------------	
	**mean ± SD**	7.26±3.7		

**Frequency of blood transfusion**	**Frequent**	32 (51.6%)	--------------------	
	**Infrequent**	30 (48.4%)		

**Iron chelation**	**Adequately chelated**	24 (38.7%)	--------------------	
	**Poorly chelated**	22 (35.5%)		
	**Not chelated**	16 (25.8%)		

**Surgical splenectomy**	**Done**	24 (38.7%)	--------------------	
	**Not done**	38 (61.3)		

**Hb level (gm/dl)**	**Range**	7–11.5	11–13.8	0.001*
	**mean± SD**	9.01±1.2	12.2 ± 0.7	

**Hematocrit value (%)**	**Range**	21–32	35–40.1	0.001*
	**mean± SD**	26.4±3.1	38.1 ± 1.2	

**Serum ferritin (ng/dl)**	**Range**	140–1900	36–97	0.001*
	**mean± SD**	890±194.3	67.5 ± 12.5	

**Serum cholesterol (mg/dl)**	**Range**	50–980	55–99	0.001*
	
	**mean±SD**	541.1±124.5	78± 13.4	

# Student t-test

* significant

**Table 2:** Comparison between patients and controls regarding CIMT(mm).

Group	Range	Mean ±SD	P value*#*
Patients(No:62)	0.4–0.6	0.48± 0.02	0.001*
Controls(No:30)	0.3–0.4	0.32± 0.05	

# Student t-test

* significant

**Table 3:** Correlations between CIMT and thalassemic patients regarding their clinical and laboratory data.

Cases	CIMT (mm) (0.46±0.02)	
	*P value*#	r^
**Age (years)**	0.001*	0.928
**Weight (kg) centile**	0.27	−0.165
**Height (cm) centile**	0.84	−0.031
**BMI (kg/m^2^)**	0.28	−0.163
**Duration since first transfusion (years)**	0.001*	0.927
**Hb level (gm/dl)**	0.07	−0.332
**Hematocrit (%)**	0.03*	−0.385
**Serum ferritin (ng/dl)**	0.001*	0.763
**Serum cholesterol (mg/dl)**	0.001*	0.830

^ Pearson Correlation

* significant

**Table 4:** CIMT in relation to clinical data in the studied cases.

Clinical parameter	Finding	Number and %	CIMT	
			Mean ± SD	P value
**Blood transfusion frequency**	Frequent (every 2 weeks)	32 (51.6%)	0.53±0.51	0.03*
	Infrequent	30 (48.4%)	0.37±0.19	
**Iron chelating agents**	Chelating	24 (38.7%)	0.39±0.04	0.02*
	Poorly chelating	22 (35.5%)	0.51±0.71	
**Splenectomy**	Done	24 (38.7%)	0.54±0.10	0.04*
	Not done	38 (61.3)	0.37±0.07	

* Significant *p*< 0.05.

**Table 5:** Risk factors of increased CIMT in B thalassemic patients.

**Risk factors of increased CIMT in B thalassemic patients** (Multiple regression analysis)			
**Variable**	Standardized Coefficients β	*P* value	Total adjustment
**Duration since 1^st^** **transfusion (years)**	0.73476	0.001	4.069
**Regularity of iron chelation**	0.444871	0.001	3.693
**Presence of splenectomy**	−0.14279	0.204	−1.307
**Hematocrit value (%)**	0.087844	0.219	1.261

## References

[1] Stamatoyannopoulos G, Majerus PW, Perlmutter RM, Varmus H (2001). The Molecular Basis of Blood Diseases.

[2] Tuzmen S, Schechter AN (2001). Genetic diseases of hemoglobin: diagnostic methods for elucidating beta-thalassemia mutations. Blood Rev.

[3] Weatherall DJ (2001). Phenotype-genotype relationships in monogenic disease: lessons from the thalassaemias. Nat Rev Genet.

[4] Hoffman R (2005). Hematology: Basic Principles and Practice.

[5] Vinchi1 Francesca, Muckenthaler Martina U, DaSilva Milene C, Balla György, Balla József, Jen Viktória Atherogenesis and iron: from epidemiology to cellular level.

[6] Borgna-Pignatti C, Rugolotto S, De Stefano P, Zhao H, Cappellini MD, Del Vecchio GC, Romeo MA, Forni GL, Gamberini MR, Ghilardi R, Piga A, Cnaan A (2004). Survival and complications in patients with thalassaemia major treated with transfusion and deferoxamine. Haematologica.

[7] Deane Colin R, Goss David, Bartram Jack, Pohl Keith RE, Height Susan E, Sibtain Naomi, Jarosz Jozef, Thein Swee Lay, Rees David C (2010). Extracranial internal carotid arterial disease in children with sickle cell anemia. Haematologica.

[8] Aggoun Y, Szezepanski I, Bonnet D (2005). Non invasive assessment of arterial stiffness and risk of atherosclerotic events in children. Pediatr Res.

[9] Järvisalo MJ, Raitakari M, Toikka JO (2004). Endothelial dysfunction and increased arterial intima-media thickness in children with type-1 diabetes. Circulation.

[10] Cheung YF (2005). Arterial Stiffness in Children and Teenagers: An Emerging Cardiovascular Risk Factor. HK J Paediatr.

[11] Cappellini Maria-Domenica, Cohen Alan, Eleftheriou Androulla, Piga Antonio, Porter John, Taher Ali (2008). Guidelines for the Clinical Management of Thalassaemia.

[12] Official 2000 centers for centers for disease control (CDC) growth charts, created by the National Center for Health Statistics (NCHS) www.cdc.gov/nchs.

[13] Cooley TB, Lee P (1925). A series of cases of splenomegaly in children with anemia and peculiar changes. Trans Am Pediatr Soc.

[14] Aessopos A, Farmakis D, Tsironi M (2007). Endothelial function and arterial stiffness in sickle-thalassemia patients. Atherosclerosis.

[15] Cheung YF, Chow PC, Chan GC, Ha SY (2006). Carotid intima-media thickness is increased and related to arterial stiffening in patients with b-thalassaemia major. Br J Haematol.

[16] Azza Tantawy AG, Amira Adly AM, Mohamed El Maaty GA, Shatha Amin AG (2009). Subclinical Atherosclerosis In Young β-thalassemia Major Patients. Hemoglobin.

[17] Gullu H, Caliskan M, Caliskan Z, Unler GK, Ermisler E, Ciftci O, Guven A, Muderrisoglu H (2013). Coronary Microvascular function, Peripheral Endothelial Function and Carotid IMT in beta-thalassemia minor. Thromb Res.

[18] Adly AA, El-Sherif NH, Ismail EA, El-Zaher YA, Farouk A, El-Refaey AM, Wahba MS (2014). Vascular Dysfunction in Patients With Young β-Thalassemia: Relation to Cardiovascular Complications and Subclinical Atherosclerosis. Clin Appl Thromb Hemost.

[19] Csmà Piccione M1, Piraino B, Zito C, Khandheria BK, Di Bella G, De Gregorio C, Oreto L, Rigoli L, Ferraù V, Salpietro CD, Carerj S (2013). Early identification of cardiovascular involvement in patients with β-thalassemia major. Am J Cardiol.

[20] Stoyanova E, Trudel M, Felfly H, Lemsaddek W, Garcia D (2012). Vascular Endothelial Dysfunction in b-Thalassemia Occurs Despite Increased eNOS Expression and Preserved Vascular Smooth Muscle Cell Reactivity to NO. PLoS ONE.

[21] Gursel O1, Kurekci AE, Tascilar E, Ileri T, Altun D, Tapan S, Kurt I, Kocaoglu M, Aydin A, Okutan V, Ozcan O (2012). Premature Atherosclerosis in Children With β-Thalassemia Major. Journal of Pediatric Hematology/Oncology.

[22] McLeod C, Fleeman N, Kirkham J, Bagust A, Boland A, Chu P, Dickson R, Dundar Y, Greenhalgh J, Modell B (2009). Deferasirox for the treatment of iron overload associated with regular blood transfusions (transfusional haemosiderosis) in patients suffering with chronic anaemia: a systematic review and economic evaluation. Health Technol Assess.

[23] Christoforidis A, Haritandi A, Tsitouridis I, Tsatra I, Tsantali H, Karyda S, Dimitriadis AS, Athanassiou-Metaxa M (2006). Correlative study of iron accumulation in liver, myocardium, and pituitary assessed with MRI inyoungthalassemic patients. J Pediatr Hematol Oncol.

[24] Papanikolaou G, Pantopoulos K (2005). Iron metabolism and toxicity. Toxicol Appl Pharmacol.

[25] Qayyum R, Schulman P (2005). Iron and atherosclerosis. Clin Cardiol.

[26] Shah SV, Alam MG (2003). Role of iron in atherosclerosis. Am J Kidney Dis.

[27] Cheung YF, Chan GC, Ha SY (2002). Arterial stiffness and endothelial function in patients with beta-thalassemia major. Circulation.

[28] Ramakrishna G, Rooke TW, Cooper LT (2003). Iron and peripheral arterial disease: revisiting the iron hypothesis in a different light. Vasc Med.

[29] Ferrara DE, Taylor WR (2005). Iron chelation and vascular function: in search of the mechanisms. Arterioscler Thromb Vasc Biol.

[30] Drueke T, Witko-Sarsat V, Massy Z, Descamps-Latscha B, Guerin AP, Marchais SJ, Gausson V, London GM (2002). Iron therapy, advanced oxidation protein products, and carotid artery intima-media thickness in end-stage renal disease. Circulation.

[31] Gaenzer H, Marschang P, Sturm W, Neumayr G, Vogel W, Patsch J, Weiss G (2002). Association between increased iron stores and impaired endothelial function in patients with hereditary hemochromatosis. J Am Coll Cardiol.

[32] deValk B, Marx JJ (1999). Iron, atherosclerosis, and ischemic heart disease. Arch Intern Med.

[33] Steinberg D, Parthasarathy S, Carew TE, Khoo JC, Witztum JL (1989). Beyond cholesterol. Modifications of low-density lipoprotein that increaseits atherogenicity. N Engl J Med.

[34] Halliwell B, Chirico S (1993). Lipid peroxidation: its mechanism, measurement, and significance. Am J Clin Nutr.

